# Sustained Intraocular Pressure Rise after the Treat and Extend Regimen at 3 Years: Aflibercept versus Ranibizumab

**DOI:** 10.1155/2020/7462098

**Published:** 2020-01-20

**Authors:** Alper Bilgic, Laurent Kodjikian, Jay Chhablani, Anand Sudhalkar, Megha Trivedi, Viraj Vasavada, Vaishali Vasavada, Shail Vasavada, Samaresh Srivastava, Deepak Bhojwani, Aditya Sudhalkar

**Affiliations:** ^1^Alpha Vision Augenzentrum, Bremerhaven, Germany; ^2^Hospices Civil de la Croix Rousse, Lyon, France; ^3^Pittsburgh Medical Centre, Pittsburgh, PA, USA; ^4^M. S. Sudhalkar Medical Research Foundation, Baroda, India; ^5^Raghudeep Eye Hospital, Ahmedabad, India

## Abstract

**Purpose:**

To determine the risk factors associated with sustained intraocular pressure (IOP) rise in patients enrolled in the treat and extend (T&E) protocol receiving aflibercept/ranibizumab therapy for 3 years.

**Design:**

Retrospective, observational chart review. *Setting*. Multicentric. *Patients*. 789 patients (1021 eyes; 602 males) enrolled in T&E using aflibercept/ranibizumab for diabetic macular edema (DME), wet age-related macular degeneration (AMD), or macular edema in retinal vein occlusion (RVO). *Intervention*. The history, examination (clinical and special investigations), and treatment records were thoroughly scrutinized. Sustained IOP rise was defined as a rise in IOP above baseline by ≥6 mmHg and/or >24 mmHg on 2 or more consecutive visits. The Wilk–Shapiro test was used for confirming normality of data. The Mantel–Haenszel test and generalized estimating equations were used to analyse multicentric data as well as to analyse data from both eyes of the same patients in the event that both eyes were under therapy. The relative risk, chi-square test (with and without Yates' correction), and univariate and multivariate analysis were used wherever appropriate. Statistical significance was set at *P* < 0.05. The primary outcome measure was the determination of risk factors for sustained IOP rise with ranibizumab/aflibercept therapy. Secondary outcome measures included determining the incidence of IOP rise (short term and sustained), visual field, and retinal nerve fibre layer (RNFL) changes.

**Results:**

The mean follow-up was 42.4 months. Male gender, South Asian ethnicity, older age, presence of AMD and vein occlusion, use of ranibizumab, higher number of injections, narrow angles, switch to bevacizumab/ranibizumab, and preexisting glaucoma were associated with sustained IOP rise. No significant visual field and RNFL changes were seen. The overall incidence was 8.91%. No patient required filtering surgery. No patient with IOP rise returned to baseline.

**Conclusion:**

IOP rise is an important consideration as the chronicity of the condition can eventually lead to glaucomatous changes in eyes with already compromised vision. Follow-ups and use of appropriate therapy can be determined correspondingly.

## 1. Introduction

Sustained intraocular pressure rise following intravitreal anti-VEGF injections is a known phenomenon, with several publications addressing this issue in part or whole [1–5]. There is a certain measure of discrepancy in reporting insofar as the potential risk factors as well as definitions of intraocular pressure (IOP) rise are concerned [6–8]. With numerous publications on the subject, it is only natural that contrasting outcomes are noted in studies conducted across the globe [1–8], the most disputed amongst risk factors for IOP rise being the number of injections administered and the treatment interval [2] between consecutive injections. When one factor in the indication, the anti-VEGF agent used, the phakic status, the anterior chamber angle status, family history of glaucoma, and other characteristics [1, 2], it is evident that the condition (IOP rise) and analysis thereof is a complex phenomenon.

Despite a plethora of literature on the subject, a recently published review [1] highlights the lack of readily identifiable risk factors for IOP rise following intravitreal injections. Additionally, a literature search on PubMed, Scopus, and the Cochrane Database on 11^th^ May 2019 using the key words “anti-VEGF agents, diabetic macular edema, retinal vein occlusion, age-related macular degeneration, choroidal neovascular membrane, intraocular pressure rise, ocular hypertension, ethnicity, anti-VEGF drug volume, short-term intraocular pressure rise, treat and extend regimen, aflibercept, ranibizumab, bevacizumab, dexamethasone implant, therapy switch, glaucoma progression, RNFL thickness, visual fields and optic disc changes” revealed a paucity of data on a comprehensive overview and hazard analysis of risk factors and IOP rise, especially between ranibizumab and aflibercept. We undertook this study with the aim of concurrently analysing all probable risk factors for sustained IOP rise following anti-VEGF injections under one complete regression model on patients enrolled under the treat and extend protocol and under follow-up for at least 3 years.

## 2. Methods

A retrospective, database search was conducted for patients who received the treat and extend protocol for wet age-related macular degeneration (wAMD), diabetic macular edema (DME), and macular edema secondary to retinal vein occlusion (RVO), and who were followed up for at least 3 years. Patients recruited had been treated at the Alphavision Augenzentrum, Bremerhaven, Germany, between January 2013 and June 2016; and the Indian centres of Raghudeep Eye Hospital, Ahmedabad; and MS Sudhalkar Medical Research Foundation, Baroda, The study adhered to the tenets of Helsinki. Informed consent about possible use of data for research had been obtained from all patients at the time of the first consultation. The chart review adhered to guidelines set out for the retrospective review process.

### 2.1. Patient Data

#### 2.1.1. Inclusion Criteria

For inclusion, patients were required to have been enrolled in the treat and extend protocol of anti-VEGF injections for one of the aforementioned conditions (diabetic macular edema, macular edema associated with vein occlusion, or age-related macular degeneration) and to have had a follow-up for 3 years at least.

#### 2.1.2. Data Chart Analysis

Data collected included a thorough history, demographics, the ethnicity of the patient, the indication for injection, the number of injections, the treatment interval, the type of anti-VEGF agent used, the volume of drug injected, therapy switch (if any), the status of the crystalline lens, the axial length, the anterior chamber angle status (per the Shaffer system; grade 2 or less was considered narrow), the relation between short-term IOP rise (measured 2 minutes after injection) and sustained IOP rise, and whether the patient was a preexisting patient of glaucoma or if the patient had a family history of glaucoma. We also noted the concentration of ranibizumab injected (0.5 mg or 0.3 mg). In India, the Drugs Controller General of India (DCGI) has approved both 0.3 mg and 0.5 mg concentrations for all three aforementioned indications, including 2 mg/0.05 ml for treatment-resistant cases [9].

#### 2.1.3. Injection Procedure

Patients received a preemptive combination of brimonidine and timolol twice daily [10], starting 24 hours prior to the day of injection followed by one drop in the morning at least 2 hours prior to the injection. Additionally, we performed ocular decompression using the technique described by Gregori and associates [11] if there was no light perception after injection on table as assessed by asking the patient to look directly into the microscope light.

The injections had been administered under antiseptic conditions and topical anesthesia using a standardized technique in the inferotemporal quadrant. Preoperative preparation was conducted with povidone-iodine. Light perception and finger counting were confirmed on table after injection. No topical/systemic antibiotics were prescribed postoperatively. The IOP was measured with the Goldmann Applanation Tonometer 2 minutes after the injection in each patient to look for short-term IOP rise. The patients were followed up after injection on days 1, 10, and 30 and later as per the treat and extend regime. The treat and extend regime was strictly followed in all patients.

#### 2.1.4. Therapy Switch and Treatment Details

Patients were advised a therapy switch based on standardized protocols. Bevacizumab could be administered to patients with neovascular AMD if therapy with ranibizumab and aflibercept was not effective; patients were required to have a minimum of 6 injections each before any switch was attempted. Patients with DME or macular edema secondary to RVO could receive the dexamethasone implant as therapy if treatment with ranibizumab and aflibercept was not effective. For DME and RVO patients, bevacizumab was permitted only if the patient showed no response to the implant or if the implant was contraindicated in a particular patient. Overall, at least 6 monthly injections of either ranibizumab or aflibercept were necessary followed by at least 6 monthly injections of the other drug before the dexamethasone implant or bevacizumab could be administered, regardless of the indication as per the protocol for treat and extend regime set by the *Deutsche Ophthalmologische Gesellschaft*, *Deutsche Retinologische Gesellschaft*, and *Berufsverband für Augenärzte* [12]. These associations also set out guidelines for patient examination (clinical examination) and follow-ups and are compulsory for receiving reimbursement [12]. Our centres in India followed the treat and extend regimen as well. It is also of note that some patients in our centres in India received 0.3 mg ranibizumab as approved of by the Drugs Controller General of India. The treat and extend protocol was, however, strictly followed as already stated. We also noted the effect of therapy switch to either bevacizumab, dexamethasone implant, or from aflibercept to ranibizumab or vice versa on IOP of patients who had been treated with either ranibizumab or aflibercept. This was done to note the influence, if any, of switching to a particular drug from a particular drug.

#### 2.1.5. Sustained IOP Rise

We defined sustained IOP rise as a rise in IOP above baseline by ≥6 mmHg and/or an IOP elevation to >24 mmHg on 2 or more consecutive visits beyond month 1 (i.e., IOP spike sustained beyond day 30) as suggested and published by Al-Abdullah and coauthors. The rise was to have been sustained for at least 6 months after first documentation of IOP rise.

#### 2.1.6. Monitoring for Glaucoma

Eyes with preexisting glaucoma received quarterly visual field assessments in accordance with the guidelines set out [12]. Nonglaucomatous eyes received annual visual field evaluations unless they developed ocular hypertension, in which case they received semiannual visual field examinations in accordance with guidelines [12]. Patients with unreliable visual fields were excluded from the analysis.

#### 2.1.7. Statistical Analysis

Descriptive statistics was used to analyze categorical variables in size (absolute frequencies) and percentage (relative frequencies). The Wilk–Shapiro test was used to confirm the normality of the data distribution. The chi-square test was used, with and without Yates' correction, wherever appropriate. The relative risk ratio was deduced for eyes receiving injections versus fellow eyes which acted as controls. The paired *t*-test was used to compare variables before and after the studied events. The Cochran–Mantel– Haenszel model for binary outcomes and generalized estimating equations were used to assimilate data from different centres as well as to analyze data from both eyes in patients who had bilateral treatment and to produce an overall result. Univariate analysis was performed to determine the association between various independent variables (such as age, indication, lens status, and number of injections) and IOP rise (dependent variable). Those variables which returned a significant association (*P*=−0.05) on univariate analysis were included in a multiple logistic regression model to determine the influence of one variable on IOP spikes after having factored in other characteristics which are known to influence the IOP. Correlation coefficients were derived to determine the strength of association between a said variable and the development of IOP rise. The results of these tests were presented as adjusted and unadjusted odds ratio, confidence intervals, and their *P* values. An odds ratio value that is greater than one indicates a higher risk of development of OHT. Fisher's exact test (with Benjamini–Hochberg adjustments of *P* value for pairwise comparisons, wherever applicable) was used to compare categorical variables between groups of various indications. A *P* value of 0.05 was considered statistically significant.

#### 2.1.8. Outcome Measures

The primary outcome measure was the determination of risk factors associated with sustained IOP rise in patients enrolled in the treat and extend protocol. The secondary outcome measures included determining the incidence of sustained IOP rise, changes in visual field defects (especially mean deviation) as noted at final follow-up from baseline, and the changes in RNFL thickness from baseline to final follow-up.

## 3. Results

### 3.1. Demographics and Characteristics

A total of 839 patients (1021 eyes; 431 males) were analyzed. The mean follow-up was 42.4 months (SD: 2.5 months; range 36–52 months).  Table 1 provides a detailed breakup of patients classified per anti-VEGF agent with reference to age, sex, indication, ethnicity, axial length, number of injections, the treatment interval, details of therapy switch, and other previously enumerated factors.

### 3.2. Transient IOP Rise

133 (13.02%) eyes were documented to have a short-term rise in IOP 2 minutes after the injection procedure at some point in time during the follow-up period. 7/133 eyes were later documented to have sustained IOP rise. 5 out of these 7 eyes had wet AMD while one each had DME and RVO. 7 eyes needed ocular decompression immediately after injection.

### 3.3. Sustained IOP Rise

Overall, 91 eyes (8.91%) demonstrated a sustained IOP rise. 14 out of 1602 untreated eyes (of the same patients) developed sustained IOP rise over the said period. All 14 eyes had dry AMD, while the fellow eye in these patients had wet AMD. Multivariate analyses demonstrated a significant association of IOP rise with male gender, younger age (<70 years), South Asian ethnicity, ranibizumab therapy, patients with AMD, vein occlusion, narrow anterior chamber angle at baseline, the number of injections administered, therapy switch to bevacizumab, and switch from aflibercept to ranibizumab. Preexisting open-angle glaucoma was also associated with sustained IOP rise, necessitating an increase in therapy. 5.33% of 1444 eyes developed ocular hypertension, giving us a relative risk of 6.95 (95% CI 3.97–12.17, *Z*-statistic 6.78, *P* < 0.0001, number needed to treat for harm 19.38; 95% CI 25.61) at one year.

### 3.4. Other Factors

Sustained IOP rise was not associated independently with the treatment interval, short-term IOP rise, axial length, female gender, therapy switch from ranibizumab to aflibercept, and choice of anti-VEGF agent prior to switch to bevacizumab. DME did not correlate well with IOP rise either and neither did a family history of glaucoma.

### 3.5. Injections and IOP Rise

IOP rise was noted after a mean of 7.2 injections with ranibizumab and 10.8 injections with aflibercept. The difference tended towards but did not attain statistical significance (*P*=0.1). The mean rise in IOP was 8.8 mmHg (range 6–19 mmHg). 43/87 patients demonstrated an IOP >28 mmHg at some point in time during the follow-up period. 10 patients were managed efficiently with monotherapy and 31 patients required 2 antiglaucoma medications while 9 required triple local therapy for IOP control. The most commonly used antiglaucoma medicine was a combination of brimonidine tartrate and timolol maleate (61 eyes).

### 3.6. Therapy Switch to the Dexamethasone Implant

134 eyes required a switch to the dexamethasone implant for DME or RVO; 78 had chronic DME and the remaining 56 had macular edema secondary to retinal vein occlusion. 4/134 patients were diagnosed to have sustained IOP rise prior to therapy switch. 14/134 eyes developed ocular hypertension secondary to dexamethasone implant injection; none of these 14 eyes had any evidence of sustained IOP rise with anti-VEGF therapy. The mean number of injections prior to switch was 14.25 (SD: 2.25) for ranibizumab and 16.14 (SD: 2.8) for aflibercept. Regardless of primary therapy (aflibercept or ranibizumab), switch to the implant was not associated with an increased propensity towards sustained IOP rise (chi-square value: 0.069. *P*=0.79; chi-square value with Yates' Correction: 0.0043. *P*=0.94).

### 3.7. Therapy Switch to Bevacizumab

87 eyes required a switch to bevacizumab therapy for wet AMD after ranibizumab/aflibercept therapy. The mean number of injections prior to the switch was 17.42 (SD 3.14) for ranibizumab and 15.46 (SD: 2.78) for aflibercept. 14/87 (16.09%) eyes developed IOP rise after a mean 6.27 injections of bevacizumab therapy.

### 3.8. Sustained IOP Rise

All patients continued with topical therapy and with injections for IOP rise during the course of follow-up. 80/87 patients required no additional therapy than what was instituted at the time the IOP rise was first detected. 7 patients required additional IOP lowering topical therapy after a mean of 5.24 injections (SD: 1.58) after topical therapy for IOP control was first instituted. 4/7 patients were under therapy with aflibercept while 3/7 patients were under therapy with ranibizumab.

### 3.9. Preexisting Glaucoma

A total of 107 eyes had preexisting glaucoma. 11/107 eyes demonstrated a worsening of IOP control during the course of follow-up and required additional therapy. 2 patients were on 3 drugs while 9 were on two drugs for glaucoma control. All 11 patients continued to do well with additional topical therapy and did not require surgical intervention.

None of the patients demonstrated visual field worsening during the follow-up period. None of the patients with preexisting glaucoma demonstrated significant visual field progression: The mean deviation for glaucomatous eyes was −2.6 ± 1.2 dB at baseline and 2.72 ± 1.06 dB at 3 years (*P*=0.09).

The mean RNFL thickness in normal patients in our analysis was 109.7 ± 7.32 microns at baseline and 108.1 ± 6.89 microns at 3 years (*P*=0.06). The mean RNFL thickness in glaucomatous eyes changed from 91.32 ± 8.11 microns at baseline to 90.02 ± 7.57 microns at 3 years (*P*=0.083). 20 patients were excluded from the analysis because of unreliable fields.

## 4. Discussion

We demonstrate an association between sustained IOP rise and the following: older age, male sex, South Asian ethnicity, narrow angles, preexisting glaucoma, >6 injections, AMD and RVO, use of ranibizumab, concentration of ranibizumab injected, and switch to ranibizumab or bevacizumab. All patients had well-controlled IOP (with local therapy) till the end of the follow-up period. None of the patients demonstrated optic nerve head changes or visual field worsening till the end of the follow-up period. RNFL thinning was demonstrated in our study but it did not reach statistically significant proportions. All patients continued to require IOP lowering medication until the end of the follow-up period. 11 patients with preexisting glaucoma required additional IOP lowering topical therapy. Not a single patient required filtering surgery till the end of the follow-up period. Patients who had a short-term IOP rise were not necessarily predisposed to develop sustained IOP rise. Patients who had sustained IOP rise with anti-VEGF therapy were not predisposed to develop IOP rise with the dexamethasone implant. Although a rise of 6 mm or 20% rise in IOP may not necessarily be detrimental to the eye in general, we chose these definitions in line with past literature for ease of interpretation, considering the fact that this may artificially inflate the number of patients who do demonstrate an IOP rise without detriment. This is so because the purpose of this study was primarily to document IOP rise and not necessarily the damage to visual fields and/or RNFL.

Male gender and South Asian ethnicity were two demographic factors associated with an increased chance of sustained IOP rise after repeated intravitreal injection. Males were represented in greater number in our study probably because of the fact that diabetes mellitus and hypertension (and their consequent complications such as macular edema and vein occlusion) were found to be higher in several of the studied ethnic groups (Turks, Indians, and Germans). Additionally, we included several ethnic groups wherein males were more likely to present for therapy as well as comply with follow-up for 3 years as was required because of cultural issues which often tend to unfortunately sideline female patients and their visual needs (Turks, Arabs, and different Indian ethnolinguistic groups). We can only speculate at this point in time that this probably has something to do with an influence of these two factors on reduced microparticle clearance of degradation products of the anti-VEGF agent through the trabecular meshwork as suggested in earlier publications. The South Asian population in general and the Indian population in particular does not seem to have a higher incidence of glaucoma, but the chances of undetected glaucoma is higher than the Caucasian population [13, 14]. However, this does not seem to be a consideration in our study since all patients were comprehensively examined prior to therapy.

Sustained IOP rise with aflibercept and ranibizumab use for AMD has been documented and studied [15]; studies have thrown up conflicting reports as regards the risk factors studied for IOP rise. Indeed, some studies do not report of any sustained IOP rise following anti-VEGF injections [1, 2, 6, 8]. The most oft studied and documented risk factors are the number of injections and the treatment interval, followed by lens status, presence of vein occlusion [2], preexisting glaucomatous disease, and angle chamber depth. Additionally, most studies that do report IOP rise are ones that follow patients over a mean of 84 weeks. This is consistent with our findings in that most patients developed an IOP spike after a mean of 7–10 injections had been administered. AMD was a risk factor for IOP rise independent of number of injections in our study. Also, the potential role of vein occlusions in IOP rise has been suggested in past analyses [1, 2].

Pretreatment [10, 11, 16] with IOP lowering medications or ocular massage has been suggested for short-term IOP rise; the long-term effect of this measure is unknown. RNFL thinning [17] has been suggested as a short-term consequence of acute IOP fluctuations. Also, vitreous reflux [18] is said to play a role in reducing immediate rise in IOP. We determine through multivariate analysis that short-term IOP rise did not correlate significantly with long-term IOP rise. A large proportion of patients in our series did not manifest an acute IOP spike. This is probably influenced by our prophylactic control of short-term IOP rise using topical therapy and globe decompression. Most studies that advise preemptive lowering of IOP did not look at the long-term consequences of these measures on sustained IOP rise [1]. This suggests that the cause for RNFL thinning as described by Martinez de la Casa and associates is probably short-term IOP rise. We did not notice significant RNFL thinning. The prophylactic use of IOP lowering medication and ocular decompression probably prevented short-term IOP fluctuations, and thus we avoided its detrimental effect on the RNFL layer.

The treatment interval in our study did not influence IOP rise unlike the findings of Mathalone et al. They reported an incidence of sustained IOP rise of 11% (comparable to our study). Overall 22 patients in their series were noted to have IOP rise. It is possible that the lower numbers (a fourth of the total number of patients we report to have sustained IOP rise) influenced the outcomes [2]. Even if we exclusively consider wet AMD patients in our series, the number of eyes under consideration is much higher than what has been reported in the study by Mathalone and associates.

The anti-VEGF agent used has generated considerable interest, with reasonably consistent findings reported from various studies. Bevacizumab [1, 2, 14, 19] has been noted by most authors to lead to sustained IOP rise followed by ranibizumab [1, 2]. Our data corroborates with past literature in that ranibizumab has a higher probability of causing sustained IOP rise when compared to aflibercept [1, 2, 19]; only one study (with insufficient numbers) reports that ranibizumab is not associated with IOP rise [8]. We also determine in our study through multivariate analysis that switching to ranibizumab or bevacizumab increases the chances of the patient developing sustained IOP rise, whereas switching to aflibercept does not [7]. This agrees well with past reports and may have something to do with the structure of ranibizumab. Also, per our analysis, switching to the dexamethasone implant after primary therapy with anti-VEGF agents does not increase the probability of IOP rise, regardless of the agent used (ranibizumab or aflibercept). This finding is somewhat in conflict with the discussion by Dedania and associates [2] based on past reports.

The outcome of research on the number of injections and its influence on long-term IOP rise is mixed; some studies suggest that this is a consideration [20], while other authors reject this theory [21, 22]. Even the average number of injections to IOP rise fluctuates between 6 [23] and 24 [24, 25].

The concentration of the injected drug, a consideration only with ranibizumab in the South Asian region in our study (given that aflibercept is only used in a dose of 0.5 mg), seems to correlate positively and independently with sustained IOP rise. A literature search using the key words “anti-VEGF agent, intraocular pressure, ranibizumab, drug volume, 0.3 mg, 0.5 mg ml, age-related macular degeneration, macular edema, sustained IOP rise, long-term IOP rise” on PubMed, Scopus, and the Cochrane Database on 11^th^ May 2019 failed to reveal any study that looks at the volume of injected ranibizumab and IOP rise; logically, a higher volume would mean a great probability of short-term IOP spikes, but we demonstrate courtesy multivariate analysis that this influences long-term IOP rise too. This has probably something to do with greater probability of trabecular meshwork obstruction with higher drug concentrations.

Whereas a narrow anterior chamber angle predisposed the patient to sustained IOP rise in our study, the axial length seemingly did not. Short-term IOP rise has been associated with short eyes and narrow chambers [23], but its influence on long-term IOP rise does not seem to have been adequately addressed.

Preexisting glaucoma and sustained IOP rise seem to have a controversial association [1, 2], with some studies reporting a strong correlation and another reporting none. Studies that report no influence of preexisting glaucoma on long-term IOP rise generally have small numbers [1]. A family history of glaucoma was reported to be a risk factor by Hoang and associates [20]; Dedania et al. [2] suggest that their exclusion of 3 patients with glaucoma might have confounded the results. Whereas one study reports the average time to IOP rise to be 39 weeks in glaucoma patients [23], we noted the time to be 25 weeks on an average in our analysis. Whereas preexisting glaucoma appeared to be a risk factor for sustained IOP rise in our study, a family history of glaucoma did not seem to predispose a patient to long-term IOP rise. Unlike the findings of Kim and associates [5], a low baseline IOP did not seem to predispose the patient to sustained IOP rise. AMD and RVO, however, were strongly associated with sustained IOP rise. Patients with AMD in our study tended to receive on an average a greater number of injections probably leading to a greater buildup of degradation microparticles and causing a rise in IOP.

None of the patients in our study received topical or peribulbar steroids; the use of the implant after therapy switch in our analysis did not seem to independently alter the IOP profile of the patient till the end of the follow-up period. Past literature reports that patients with a history of ocular or systemic corticosteroid use had a rapid and greater increase in IOP [2, 20]. Our prophylactic treatment probably influenced this. The hypothesis that alteration of trabecular outflow facility with steroid use may influence sustained IOP rise after anti-VEGF injections probably needs further evaluation.

The extreme variations in reports on long-term IOP rise along with the risk factors responsible for it as reported in literature are testimony to the complexity of this disease process [1, 2, 18–22, 24–26]. Studies vary in their structure, number, indications, inclusion and exclusion of certain groups of patients (glaucomatous eyes, for instance), and their definitions of IOP rise [1, 2]. The current study is an attempt to compile, as comprehensively as possible, the overall data on potential risk factors (based on past literature) for sustained IOP rise following intravitreal injections and their outcomes on visual fields, optic nerve head changes, and RNFL thickness. RNFL thickness has not shown to vary significantly in literature published earlier [27]. Unlike most reports on dexamethasone implant induced transient ocular hypertension [28, 29], the rise in IOP with anti-VEGF agents seems to be chronic, sustained, thereby suggesting a higher chance of progression to glaucomatous changes, the lower incidence overall of ocular hypertension notwithstanding. We attempt to homogenize the data as much as possible in that we look exclusively at patients enrolled for the treat and extend protocol. On the other hand, the multicentric data ensures a composite ethnic assimilation and helps look at the influence of ethnicity on IOP rise. It also provides us an opportunity to look at lower ranibizumab injection volumes as protection against IOP rise. We provide data over a 3-year follow-up period, ensuring adequacy in terms of time and sufficient number of injections for analyses. We report on therapy switch to four of the most commonly used agents and their influence on IOP rise. We look at short-term IOP rise and measures to control IOP spikes in the immediate postinjection period, and we monitor patients for glaucomatous changes over the three-year follow-up period.

Our study is not without limitations: the retrospective nature and hence missed follow-ups, the lack of a control group for injections, the multicentric model (albeit adjusted statically) and perhaps the lack of a clear explanation for gender and ethnic susceptibilities, and the primacy of ranibizumab over aflibercept in IOP rise. Notwithstanding, we present several features of interest, a majority of which have already been elaborated above. Additionally, we compare head to head two FDA approved anti-VEGF agents and compile data on the treat and extend regime, the most recommended and currently the most commonly used posology, especially in insured markets in Europe, Asia, and probably the Americas, and we attempt to identify the populace most at risk for developing ocular hypertension. The compliance mandated by the insurance companies in terms of follow-up as well as our strict outreach program to avoid attrition and missed follow-ups help us draw meaningful conclusions from our data and eliminate to a large extent the fallacies of any retrospective analysis.

From our analysis, we hypothesize that the association of sustained IOP rise with age, narrow angles, greater number of injections, the volume of ranibizumab injected, and bevacizumab and ranibizumab suggests that a higher buildup of microdegradation products in the trabecular meshwork leads to sustained IOP rise. The association of AMD with sustained IOP rise is probably a pointer towards an overall degenerative process affecting the eye, a hypothesis that finds support in the fact that 14 control eyes developed ocular hypertension and all had dry AMD. Vein occlusions are closely associated with glaucoma, a pointer again to degenerative processes affecting the trabecular meshwork or dysfunctional trabecular meshwork. Whether circulating anti-VEGF molecules eventually reached the control eye is as of now unknown. The role of gender and ethnicity in trabecular meshwork function along with the proposed hypothesis needs further study. The differences in structure between aflibercept and ranibizumab may account for the difference in incidence of IOP rise too. The literature supports the role of trabecular alteration secondary to multiple injections, trabecular congestion due to antibodies, silicone microdroplets, or protein aggregation with bevacizumab and a chronic trabeculitis or a trabecular autoimmune reaction [30]. These factors seem to cause IOP rise in these patients.

To conclude, younger age, male sex, South Asian ethnicity, narrow angles, preexisting glaucoma, >6 injections, presence of AMD and RVO, use of ranibizumab, concentration of ranibizumab injected, and switch to ranibizumab or bevacizumab are independent risk factors for IOP spikes in patients who received either ranibizumab or aflibercept per the treat and extend regime for patients with AMD, DME, or RVO. Patients with the aforementioned characteristics will probably benefit with preemptive IOP lowering therapy, a close follow-up, and regular assessment for glaucomatous changes. The severity of the treat and extend regime might actually be beneficial in ensuring that these patients do not progress to develop glaucoma and end up with worse visual function.

## Figures and Tables

**Table 1:** Univariate and multivariate analysis of characteristics associated with IOP spikes after antivascular endothelial growth factor agents in the treat and extend regimen.

Characteristics	Univariate analysis			Multivariate analysis		
	Odds ratio	95% CI	*P* value	Adjusted OR	CI	*P* value
Age (years)						
>70	Ref					
<70	3.34	1.32–5.72	0.012	3.72	1.72–4.63	0.015

Gender						
Female	Ref					
Male	2.83	1.02–4.76	0.024	2.94	1.1–4.89	0.018

Lens status						
Pseudophakic	Ref					
Phakic	1.04	0.84–1.46	0.21			

Etiology						
*DME*	Ref					
*AMD*	3.31	1.34–4.28	0.01	2.40	1.43–4.57	0.009
*RVO*	1.47	1.23–2.12	0.28			

Anti-VEGF agent						
Aflibercept	Ref					
Ranibizumab	6.62	2.95–8.89	0.001	5.85	2.07–7.24	0.001

Ac angle						
TM seen	Ref					
TM not seen	4.27	3.17–5.94	0.002	3.15	1.87–5.34	0.017

Ethnicity						
German	Ref					
South Asian	2.89	1.76–5.13	0.023	3.14	1.87–4.32	0.013
Turkish	1.57	1.33–2.19	0.22			
Arab	1.42	1.32–1.89	0.19			

Short-term IOP rise						
No	Ref					
Yes	2.31	2.12–4.33	0.24			

Baseline IOP						
<14 mm Hg	Ref					
14 mm or higher	2.17	1.27–5.32	0.12			

Ranibizumab volume (ml)						
0.03						
0.05	4.31	2.18–6.75	0.001	3.78	1.32–5.75	0.001

Treatment interval (weeks)						
4	Ref					
>4	2.31	2.09–4.12	0.11			

Number of injections						
3 or less	Ref					
3–6	3.35	1.67–3.87	0.07			
>6	3.24	2.09–5.08	0.012	4.11	1.83–5.39	0.001

Therapy switch						
To aflibercept	Ref					
To ranibizumab	4.13	2.29–6.03	0.003	3.78	2.10–4.78	0.002
To DEXI	3.11	2.87–5.4	0.09			
To avastin	5.12	2.56–7.25	0.011	4.55	2.17–6.78	0.002

Glaucoma						
No glaucoma	Ref					
Preexisting	3.11	2.78–5.97	0.013	4.13	3.12–5.89	0.001

F/H glaucoma						
No	Ref					
Yes	1.57	1.33–4.21	0.14			

Axial length (mm)						
23.0–25.0	Ref					
<23.0	2.34	1.42–5.22	0.13			
>25.0	1.85	1.2–3.98	0.10			

CI: confidence interval, DME: diabetic macular edema, OR: odds ratio, *P*=*p* value, AMD: age-related macular degeneration, RVO: retinal vein occlusion, TM: trabecular meshwork, DEXI: dexamethasone implant. “Ref” is short for “Reference for statistical comparison of independent variables with more than one possible outcome during multivariate analysis.

## Data Availability

Data pertaining to this manuscript will be made available on request.
